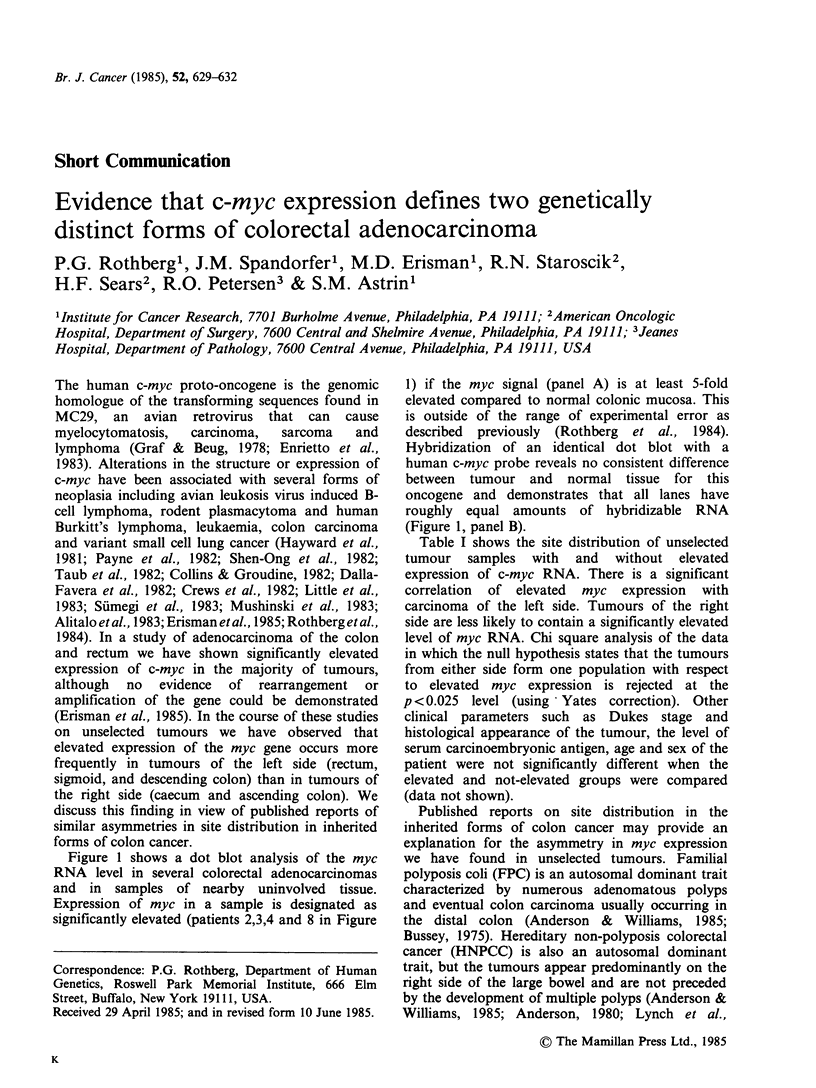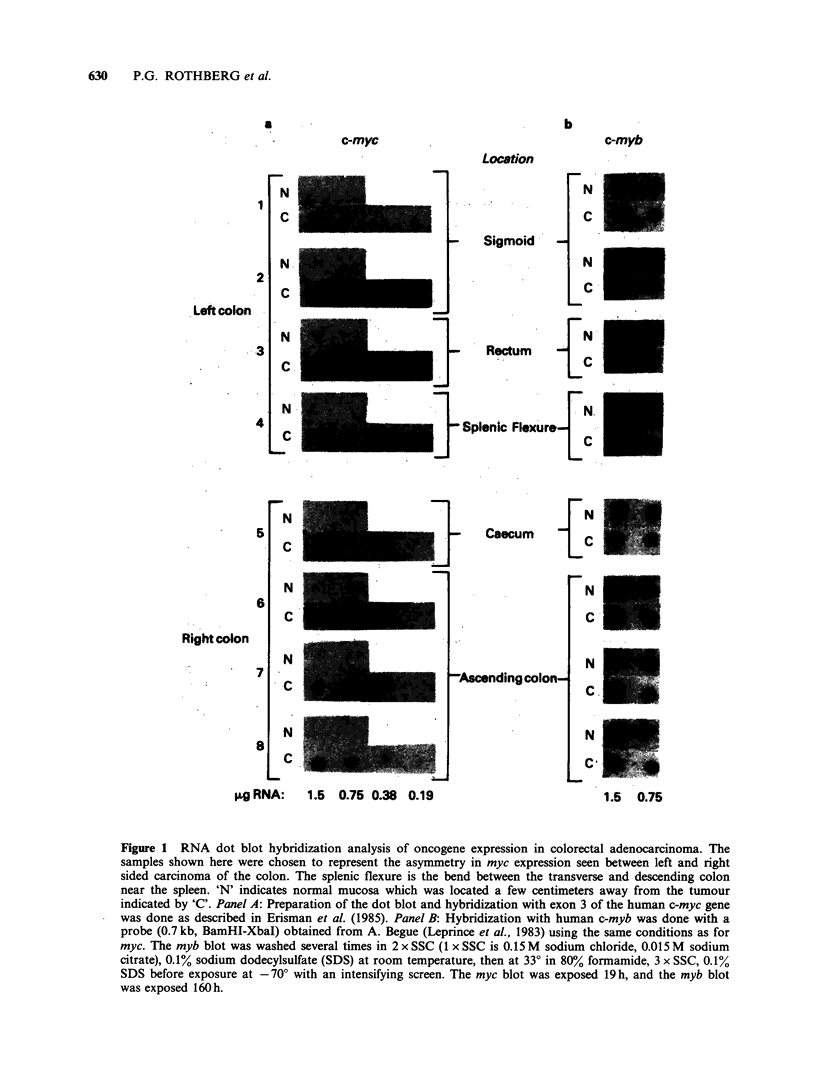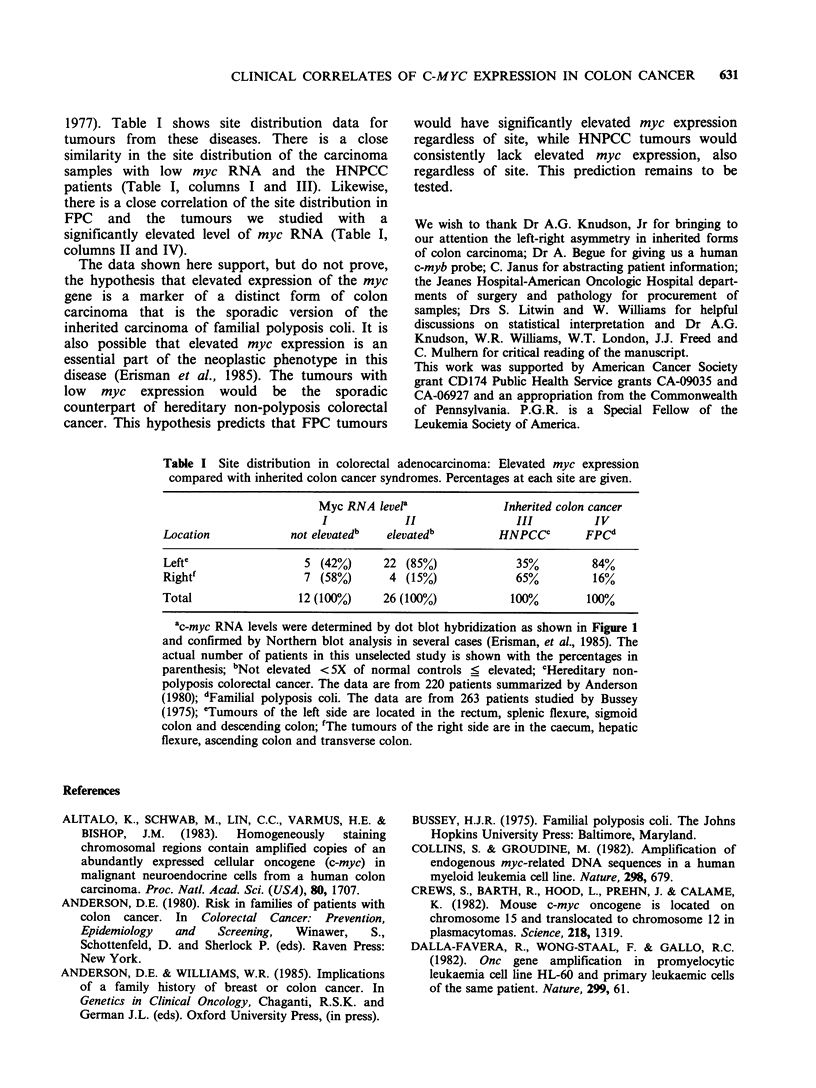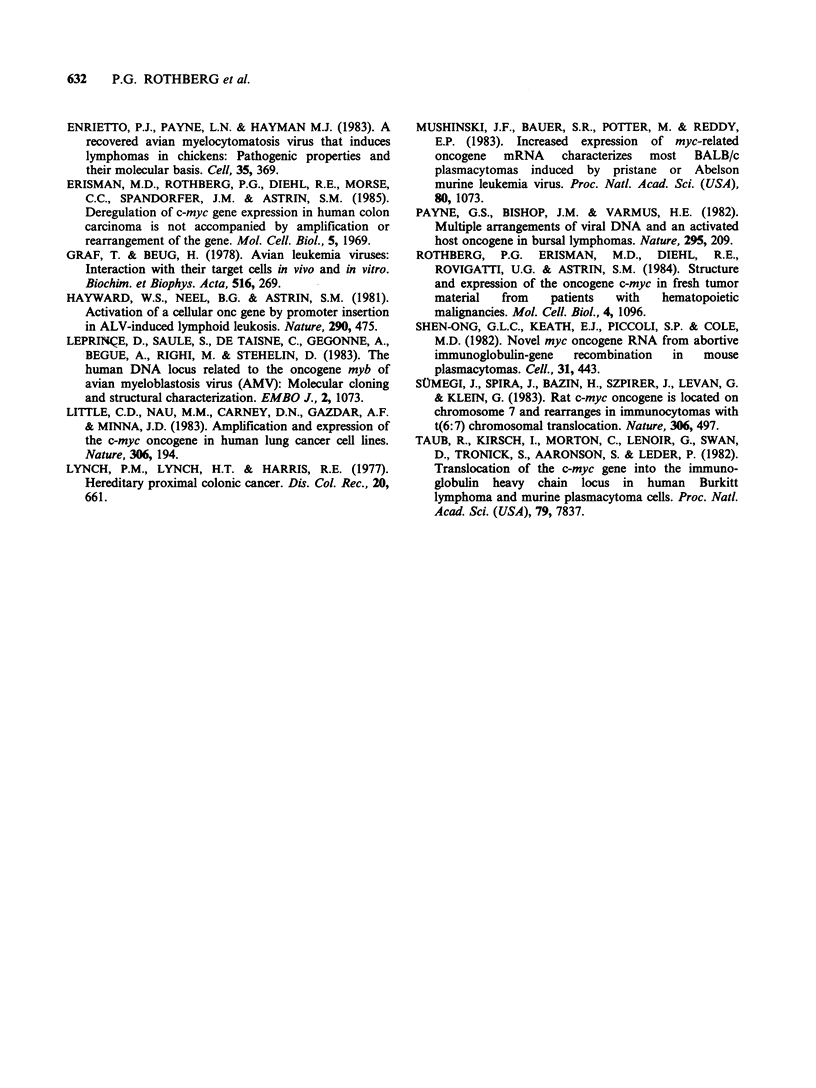# Evidence that c-myc expression defines two genetically distinct forms of colorectal adenocarcinoma.

**DOI:** 10.1038/bjc.1985.237

**Published:** 1985-10

**Authors:** P. G. Rothberg, J. M. Spandorfer, M. D. Erisman, R. N. Staroscik, H. F. Sears, R. O. Petersen, S. M. Astrin

## Abstract

**Images:**


*Br. J. Cancer* (1985), **52**, 629–632

### Short Communication

### Evidence that c-*myc* expression defines two genetically distinct forms of colorectal adenocarcinoma

P.G. Rothberg[1], J.M. Spandorfer[1], M.D. Erisman[1], R.N. Staroscik[2], H.F. Sears[2], R.O. Petersen[3] & S.M. Astrin[1]

[1]*Institute for Cancer Research, 7701 Burholme Avenue, Philadelphia, PA 19111;* [2]*American Oncologic Hospital, Department of Surgery, 7600 Central and Shelmire Avenue, Philadelphia, PA 19111;* [3]*Jeanes Hospital, Department of Pathology, 7600 Central Avenue, Philadelphia, PA 19111, USA*

The human c-*myc* proto-oncogene is the genomic homologue of the transforming sequences found in MC29, an avian retrovirus that can cause myelocytomatosis, carcinoma, sarcoma and lymphoma (Graf & Beug, 1978; Enrietto *et al.*, 1983). Alterations in the structure or expression of c-*myc* have been associated with several forms of neoplasia including avian leukosis virus induced B-cell lymphoma, rodent plasmacytoma and human Burkitt's lymphoma, leukaemia, colon carcinoma and variant small cell lung cancer (Hayward *et al.*, 1981; Payne *et al.*, 1982; Shen-Ong *et al.*, 1982; Taub *et al.*, 1982; Collins & Groudine, 1982; Dalla-Favera *et al.*, 1982; Crews *et al.*, 1982; Little *et al.*, 1983; Sümegi *et al.*, 1983; Mushinski *et al.*, 1983; Alitalo *et al.*, 1983; Erisman *et al.*, 1985; Rothberg *et al.*, 1984). In a study of adenocarcinoma of the colon and rectum we have shown significantly elevated expression of c-*myc* in the majority of tumours, although no evidence of rearrangement or amplification of the gene could be demonstrated (Erisman *et al.*, 1985). In the course of these studies on unselected tumours we have observed that elevated expression of the *myc* gene occurs more frequently in tumours of the left side (rectum, sigmoid, and descending colon) than in tumours of the right side (caecum and ascending colon). We discuss this finding in view of published reports of similar asymmetries in site distribution in inherited forms of colon cancer.

Figure 1 shows a dot blot analysis of the *myc* RNA level in several colorectal adenocarcinomas and in samples of nearby uninvolved tissue. Expression of *myc* in a sample is designated as significantly elevated (patients 2,3,4 and 8 in Figure 1) if the *myc* signal (panel A) is at least 5-fold elevated compared to normal colonic mucosa. This is outside of the range of experimental error as described previously (Rothberg *et al.*, 1984). Hybridization of an identical dot blot with a human c-*myc* probe reveals no consistent difference between tumour and normal tissue for this oncogene and demonstrates that all lanes have roughly equal amounts of hybridizable RNA (Figure 1, panel B).

Table I shows the site distribution of unselected tumour samples with and without elevated expression of c-*myc* RNA. There is a significant correlation of elevated *myc* expression with carcinoma of the left side. Tumours of the right side are less likely to contain a significantly elevated level of *myc* RNA. Chi square analysis of the data in which the null hypothesis states that the tumours from either side form one population with respect to elevated *myc* expression is rejected at the $p<0.025$ level (using Yates correction). Other clinical parameters such as Dukes stage and histological appearance of the tumour, the level of serum carcinoembryonic antigen, age and sex of the patient were not significantly different when the elevated and not-elevated groups were compared (data not shown).

Published reports on site distribution in the inherited forms of colon cancer may provide an explanation for the asymmetry in *myc* expression we have found in unselected tumours. Familial polyposis coli (FPC) is an autosomal dominant trait characterized by numerous adenomatous polyps and eventual colon carcinoma usually occurring in the distal colon (Anderson & Williams, 1985; Bussey, 1975). Hereditary non-polyposis colorectal cancer (HNPCC) is also an autosomal dominant trait, but the tumours appear predominantly on the right side of the large bowel and are not preceded by the development of multiple polyps (Anderson & Williams, 1985; Anderson, 1980; Lynch *et al.*,

Correspondence: P.G. Rothberg, Department of Human Genetics, Roswell Park Memorial Institute, 666 Elm Street, Buffalo, New York 19111, USA.
Received 29 April 1985; and in revised form 10 June 1985.

© The Mamillan Press Ltd., 1985

**Figure 1** RNA dot blot hybridization analysis of oncogene expression in colorectal adenocarcinoma. The samples shown here were chosen to represent the asymmetry in *myc* expression seen between left and right sided carcinoma of the colon. The splenic flexure is the bend between the transverse and descending colon near the spleen. 'N' indicates normal mucosa which was located a few centimeters away from the tumour indicated by 'C'. *Panel A*: Preparation of the dot blot and hybridization with exon 3 of the human c-*myc* gene was done as described in Erisman *et al.* (1985). *Panel B*: Hybridization with human c-*myb* was done with a probe (0.7 kb, BamHI-XbaI) obtained from A. Begue (Leprince *et al.*, 1983) using the same conditions as for *myc*. The *myb* blot was washed several times in 2 × SSC (1 × SSC is 0.15 M sodium chloride, 0.015 M sodium citrate), 0.1% sodium dodecylsulfate (SDS) at room temperature, then at 33° in 80% formamide, 3 × SSC, 0.1% SDS before exposure at −70° with an intensifying screen. The *myc* blot was exposed 19 h, and the *myb* blot was exposed 160 h.

1977). Table I shows site distribution data for tumours from these diseases. There is a close similarity in the site distribution of the carcinoma samples with low *myc* RNA and the HNPCC patients (Table I, columns I and III). Likewise, there is a close correlation of the site distribution in FPC and the tumours we studied with a significantly elevated level of *myc* RNA (Table I, columns II and IV).

The data shown here support, but do not prove, the hypothesis that elevated expression of the *myc* gene is a marker of a distinct form of colon carcinoma that is the sporadic version of the inherited carcinoma of familial polyposis coli. It is also possible that elevated *myc* expression is an essential part of the neoplastic phenotype in this disease (Erisman *et al.*, 1985). The tumours with low *myc* expression would be the sporadic counterpart of hereditary non-polyposis colorectal cancer. This hypothesis predicts that FPC tumours would have significantly elevated *myc* expression regardless of site, while HNPCC tumours would consistently lack elevated *myc* expression, also regardless of site. This prediction remains to be tested.

We wish to thank Dr A.G. Knudson, Jr for bringing to our attention the left-right asymmetry in inherited forms of colon carcinoma; Dr A. Begue for giving us a human c-*myb* probe; C. Janus for abstracting patient information; the Jeanes Hospital-American Oncologic Hospital departments of surgery and pathology for procurement of samples; Drs S. Litwin and W. Williams for helpful discussions on statistical interpretation and Dr A.G. Knudson, W.R. Williams, W.T. London, J.J. Freed and C. Mulhern for critical reading of the manuscript.
This work was supported by American Cancer Society grant CD174 Public Health Service grants CA-09035 and CA-06927 and an appropriation from the Commonwealth of Pennsylvania. P.G.R. is a Special Fellow of the Leukemia Society of America.

**Table I** Site distribution in colorectal adenocarcinoma: Elevated *myc* expression compared with inherited colon cancer syndromes. Percentages at each site are given.

| | Myc *RNA level*[a] | | *Inherited colon cancer* | |
|---|---|---|---|---|
| | *I* | *II* | *III* | *IV* |
| *Location* | *not elevated*[b] | *elevated*[b] | *HNPCC*[c] | *FPC*[d] |
| Left[e] | 5 (42%) | 22 (85%) | 35% | 84% |
| Right[f] | 7 (58%) | 4 (15%) | 65% | 16% |
| Total | 12 (100%) | 26 (100%) | 100% | 100% |

[a]c-*myc* RNA levels were determined by dot blot hybridization as shown in **Figure 1** and confirmed by Northern blot analysis in several cases (Erisman, *et al.*, 1985). The actual number of patients in this unselected study is shown with the percentages in parenthesis; [b]Not elevated $<5X$ of normal controls $\leq$ elevated; [c]Hereditary non-polyposis colorectal cancer. The data are from 220 patients summarized by Anderson (1980); [d]Familial polyposis coli. The data are from 263 patients studied by Bussey (1975); [e]Tumours of the left side are located in the rectum, splenic flexure, sigmoid colon and descending colon; [f]The tumours of the right side are in the caecum, hepatic flexure, ascending colon and transverse colon.

### References

ALITALO, K., SCHWAB, M., LIN, C.C., VARMUS, H.E. & BISHOP, J.M. (1983). Homogeneously staining chromosomal regions contain amplified copies of an abundantly expressed cellular oncogene (c-*myc*) in malignant neuroendocrine cells from a human colon carcinoma. *Proc. Natl. Acad. Sci.* (*USA*), **80**, 1707.

ANDERSON, D.E. (1980). Risk in families of patients with colon cancer. In *Colorectal Cancer: Prevention, Epidemiology and Screening,* Winawer, S., Schottenfeld, D. and Sherlock P. (eds). Raven Press: New York.

ANDERSON, D.E. & WILLIAMS, W.R. (1985). Implications of a family history of breast or colon cancer. In *Genetics in Clinical Oncology,* Chaganti, R.S.K. and German J.L. (eds). Oxford University Press, (in press).

BUSSEY, H.J.R. (1975). Familial polyposis coli. The Johns Hopkins University Press: Baltimore, Maryland.

COLLINS, S. & GROUDINE, M. (1982). Amplification of endogenous *myc*-related DNA sequences in a human myeloid leukemia cell line. *Nature,* **298,** 679.

CREWS, S., BARTH, R., HOOD, L., PREHN, J. & CALAME, K. (1982). Mouse c-*myc* oncogene is located on chromosome 15 and translocated to chromosome 12 in plasmacytomas. *Science,* **218,** 1319.

DALLA-FAVERA, R., WONG-STAAL, F. & GALLO, R.C. (1982). *Onc* gene amplification in promyelocytic leukaemia cell line HL-60 and primary leukaemic cells of the same patient. *Nature,* **299,** 61.

ENRIETTO, P.J., PAYNE, L.N. & HAYMAN M.J. (1983). A recovered avian myelocytomatosis virus that induces lymphomas in chickens: Pathogenic properties and their molecular basis. *Cell*, **35**, 369.

ERISMAN, M.D., ROTHBERG, P.G., DIEHL, R.E., MORSE, C.C., SPANDORFER, J.M. & ASTRIN, S.M. (1985). Deregulation of c-*myc* gene expression in human colon carcinoma is not accompanied by amplification or rearrangement of the gene. *Mol. Cell. Biol.*, **5**, 1969.

GRAF, T. & BEUG, H. (1978). Avian leukemia viruses: Interaction with their target cells *in vivo* and *in vitro*. *Biochim. et Biophys. Acta*, **516**, 269.

HAYWARD, W.S., NEEL, B.G. & ASTRIN, S.M. (1981). Activation of a cellular onc gene by promoter insertion in ALV-induced lymphoid leukosis. *Nature*, **290**, 475.

LEPRINCE, D., SAULE, S., DE TAISNE, C., GEGONNE, A., BEGUE, A., RIGHI, M. & STEHELIN, D. (1983). The human DNA locus related to the oncogene *myb* of avian myeloblastosis virus (AMV): Molecular cloning and structural characterization. *EMBO J.*, **2**, 1073.

LITTLE, C.D., NAU, M.M., CARNEY, D.N., GAZDAR, A.F. & MINNA, J.D. (1983). Amplification and expression of the c-*myc* oncogene in human lung cancer cell lines. *Nature*, **306**, 194.

LYNCH, P.M., LYNCH, H.T. & HARRIS, R.E. (1977). Hereditary proximal colonic cancer. *Dis. Col. Rec.*, **20**, 661.

MUSHINSKI, J.F., BAUER, S.R., POTTER, M. & REDDY, E.P. (1983). Increased expression of *myc*-related oncogene mRNA characterizes most BALB/c plasmacytomas induced by pristane or Abelson murine leukemia virus. *Proc. Natl. Acad. Sci.* (*USA*), **80**, 1073.

PAYNE, G.S., BISHOP, J.M. & VARMUS, H.E. (1982). Multiple arrangements of viral DNA and an activated host oncogene in bursal lymphomas. *Nature*, **295**, 209.

ROTHBERG, P.G. ERISMAN, M.D., DIEHL, R.E., ROVIGATTI, U.G. & ASTRIN, S.M. (1984). Structure and expression of the oncogene c-*myc* in fresh tumor material from patients with hematopoietic malignancies. *Mol. Cell. Biol.*, **4**, 1096.

SHEN-ONG, G.L.C., KEATH, E.J., PICCOLI, S.P. & COLE, M.D. (1982). Novel *myc* oncogene RNA from abortive immunoglobulin-gene recombination in mouse plasmacytomas. *Cell.*, **31**, 443.

SÜMEGI, J., SPIRA, J., BAZIN, H., SZPIRER, J., LEVAN, G. & KLEIN, G. (1983). Rat c-*myc* oncogene is located on chromosome 7 and rearranges in immunocytomas with t(6:7) chromosomal translocation. *Nature*, **306**, 497.

TAUB, R., KIRSCH, I., MORTON, C., LENOIR, G., SWAN, D., TRONICK, S., AARONSON, S. & LEDER, P. (1982). Translocation of the c-*myc* gene into the immunoglobulin heavy chain locus in human Burkitt lymphoma and murine plasmacytoma cells. *Proc. Natl. Acad. Sci.* (*USA*), **79**, 7837.