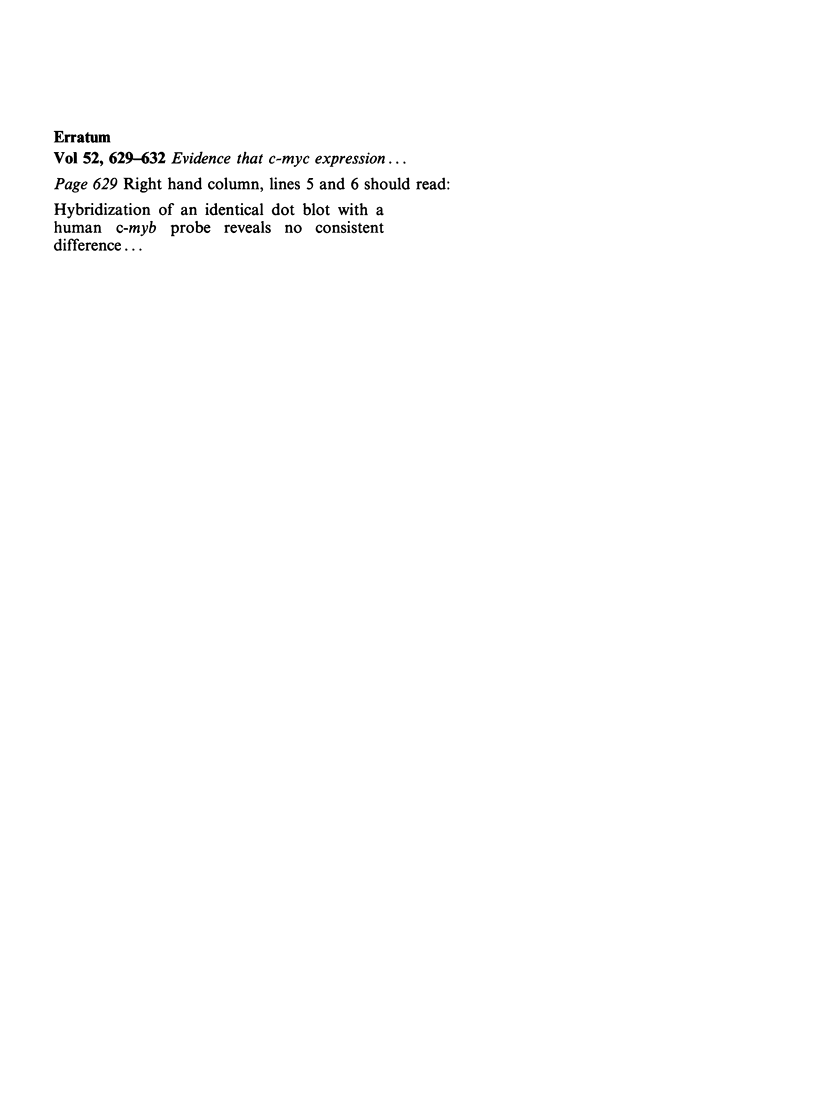# Erratum

**Published:** 1986-02

**Authors:** 


**Erratum**

**Vol 52, 629–632** *Evidence that c-myc expression…*

*Page 629* Right hand column, lines 5 and 6 should read:

Hybridization of an identical dot blot with a human c-*myb* probe reveals no consistent difference…